# Pathogenic mutations in neurofibromin identifies a leucine-rich domain regulating glioma cell invasiveness

**DOI:** 10.1038/s41388-019-0809-3

**Published:** 2019-04-09

**Authors:** Siti Farah Bte Fadhlullah, Nurashikin Bte Abdul Halim, Jacqueline Y. T. Yeo, Rachel L. Y. Ho, Phoebe Um, Beng Ti Ang, Carol Tang, Wai H. Ng, David M. Virshup, Ivy A. W. Ho

**Affiliations:** 1 Molecular Neurotherapeutics Laboratory, National Neuroscience Institute, Singapore, 308433 Singapore; 2 University of Pennsylvania, Philadelphia, PA 19104 USA; 3 Department of Neurosurgery, National Neuroscience Institute, Singapore, 308433 Singapore; 4 Department of Physiology, Yong Loo Lin School of Medicine, National University of Singapore, Singapore, 119228 Singapore; 5 Singapore Institute for Clinical Sciences, A*STAR, Singapore, 117609 Singapore; 6 Duke-NUS Medical School, Singapore, 169857 Singapore; 7 Department of Research, National Neuroscience Institute, Singapore, 308433 Singapore; 8 Program in Cancer and Stem Cell Biology, Duke-NUS Medical School, Singapore, 169857 Singapore; 9 Division of Cellular and Molecular Research, National Cancer Centre, Singapore, 169610 Singapore; 10 Department of Pediatrics, Duke University School of Medicine, Durham, NC 27703 USA; 11 Present Address: Lucence Diagnostics Pte Ltd., Singapore, Singapore

**Keywords:** Cancer stem cells, CNS cancer, RHO signalling, Metastasis

## Abstract

Glioblastoma (GBM) is the most aggressive tumor of the brain. *NF1*, a tumor suppressor gene and RAS-GTPase, is one of the highly mutated genes in GBM. Dysregulated *NF1* expression promotes cell invasion, proliferation, and tumorigenesis. Loss of *NF1* expression in glioblastoma is associated with increased aggressiveness of the tumor. Here, we show that *NF1*-loss in patient-derived glioma cells using shRNA increases self-renewal, heightens cell invasion, and promotes mesenchymal subtype and epithelial mesenchymal transition-specific gene expression that enhances tumorigenesis. The neurofibromin protein contains at least four major domains, with the GAP-related domain being the most well-studied. In this study, we report that the leucine-rich domain (LRD) of neurofibromin inhibits invasion of human glioblastoma cells without affecting their proliferation. Moreover, under conditions tested, the NF1-LRD fails to hydrolyze Ras-GTP to Ras-GDP, suggesting that its suppressive function is independent of Ras signaling. We further demonstrate that rare variants within the NF1-LRD domain found in a subset of the patients are pathogenic and reduce NF1-LRD’s invasion suppressive function. Taken together, our results show, for the first time, that NF1-LRD inhibits glioma invasion, and provides evidence of a previously unrecognized function of NF1-LRD in glioma biology.

## Introduction

Glioblastoma (GBM) is the most malignant tumor of the brain with patients having a median survival of less than 15 months [1, 2]. Current standard therapy after initial diagnosis, includes maximal surgical debulking followed by adjuvant temozolomide administration and radiation therapy. However, due to the highly infiltrative and heterogeneous nature of the tumor cells, invading cells render complete surgical resection impossible, making recurrence of tumor growth an intractable clinical issue [3, 4].

The tumor suppressor gene *NF1* is mutated or suppressed in a variety of sporadic cancers such as neuroblastoma, melanoma, and nonsmall-cell lung cancer [5–9]. Notably, *NF1* is mutated or deregulated in approximately 13% of GBM, ranking it the third most frequently somatically mutated gene sequence in GBM [10–12]. These mutations, include nonsense mutations, splice site mutations, frameshift indels, and missense mutations. In GBM, *NF1* loss or mutation is observed primarily in the more aggressive mesenchymal subtype, suggesting its role as a driver of mesenchymal transition [13–17].

Neurofibromin, the protein product of the *NF1* gene, is a RAS GTPase-activating protein (RAS-GAP) that negatively regulates Ras activity by catalyzing the hydrolysis of RAS-GTP [18, 19]. Deregulated Ras expression thus results in activation of downstream proteins and transcription factors, some of which are associated with the epithelial–mesenchymal transition (EMT). EMT-associated transcription factors such as SNAIL (SNAI1), SLUG (SNAI2), Twist Family BHLH Transcription Factor (TWIST)-1, Zinc Finger E-Box Binding Homeobox (ZEB) have been shown to be upregulated in malignant peripheral nerve sheath tumor (MPNST) deficient for neurofibromin [20, 21]. Loss of *NF1* also triggers the activation of multiple signaling pathways, including Rho/Rho associated coiled-coil containing protein Kinase (ROCK)/LIM domain kinase (LIMK) signaling that promotes changes in actin cytoskeleton, thereby regulating cellular motility [22]. LIMK2 is a microtubule-associated protein that enhances microtubule stability when it is unphosphorylated. It has been suggested that the binding of SEC14-Plekstrin Homology (PH) domain of neurofibromin to LIMK2 prevents activation of LIMK2 by ROCK, thereby resulting in reduced actin polymerization and inhibition of cell invasion. On the other hand, in *NF1*-null cells, LIMK2 is hyperphosphorylated by ROCK, leading to enhanced cell migration and invasion [22]. Downregulation of neurofibromin not only encourages EMT transition, it also promotes intrinsic resistance to inhibitors along the Ras-RAF-MEK-ERK pathway by RAS activation [8, 23, 24]. Mutation in the *BRAF* variant V600E, upregulation of receptor tyrosine kinases (RTK) such as epidermal growth factor receptor or activation of mitogen activated protein kinase (MEK) are some of the mechanisms of resistance in *NF1* mutant or deficient tumors [8, 10, 25–27]. Recent studies found that MEK inhibitor (MEKi), such as Sorafenib, are effective in *NF1*-associated MPNST [28]. However, despite its antitumor efficacy in MPNST in vitro [28–30], it fails to deliver therapeutic benefit in the clinics [31]. On the other hand, the MEKi Selumetinib, appears to be beneficial for children with neurofibromatosis 1 (NF1) [32]. The limited efficacy of MEKi suggested that single agent MEKi is insufficient to combat cancer growth, and MEKi may require other combinations such as mTORC1/2 inhibitors to be effective [33].

Neurofibromin has multiple domains, with the GAP-related domain (GRD) being the most well-studied. Other key functional domains involved in neurofibromin function, include the leucine-rich domain (LRD), the cysteine-serine rich domain (CSRD), and the C-terminal domain (CTD) [34, 35]. Each domain interacts with various effectors to modulate cellular functions. For example, binding of GRD to sprouty-related EVH1 domain-containing protein 1 (Spred1) inhibits Spred1 function and localizes GRD to the membrane to inactivate membrane-anchored Ras [36]. The CTD domain, on the other hand, regulates neurite outgrowth and dendritic filopodia formation through its interaction with collapsin response mediator proteins (CRMP)-2 and -4 [37, 38] and syndecan-2, respectively [39]. CTD also mediates cell adhesion through its binding with focal adhesion kinase [40]. Both CTD and CSRD interact with dimethylarginine dimethylaminohydrolase 1 (DDAH1), which degrades the endogenous nitric oxide inhibitor asymmetric dimethylarginine (ADMA) responsible for regulating cell proliferation [35]. The LRD domain spans amino acids (aa) 1579–1971 (UniProtKB-P21359; neurofibromin isoform 2) and consists of a known SEC14-PH domain and a portion of the HEAT-like repeats (HLR) or the structurally related Armadillo (ARM) superfamily [35, 41]. Based on structural and biochemical analysis, the bipartite SEC14-PH domain was shown to bind to phospholipids [42, 43]. The NF1-LRD domain was first shown by Wang et al. to regulate dendritic spine formation through its interaction with valosin-containing protein [41]. They further demonstrated that c.4759_4761delTAT; Y1587(delta) mutation observed in the NF1-LRD domain almost completely abolished this interaction.

In the current study, we show that the NF1-LRD plays a critical role in suppressing glioma cell invasion both in vitro and in an orthotopic glioma mouse model. We further demonstrate that pathogenic mutations within this domain abolish its invasion suppression function, suggesting a role for the NF1-LRD against tumor metastasis and invasion.

## Results

### Inverse relationship between NF1-expression levels and invasive aggressiveness

**Fig. 1:**
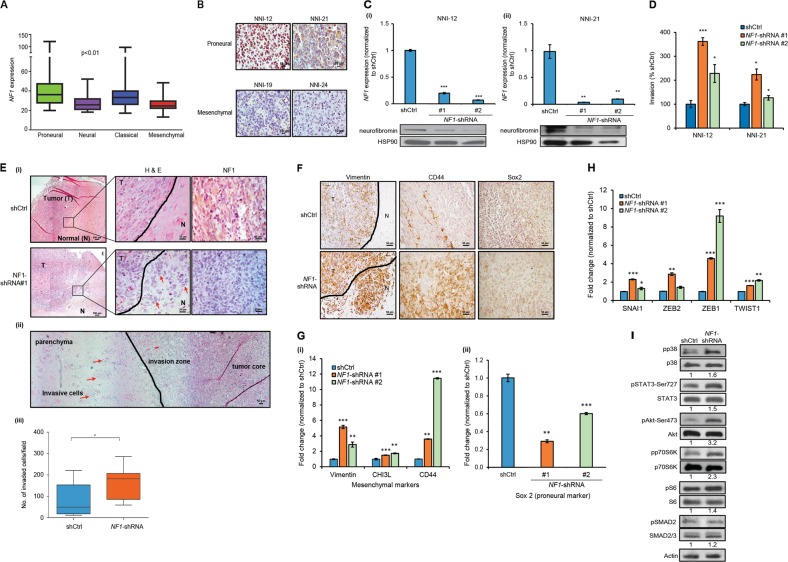
*NF1* loss promotes GPCs invasion in vitro and in vivo. **a** *NF1* mRNA expression in GBM subtypes. One-way ANOVA with Tukey’s test was used to analyze statistical significance, *p < 0.01. Data are represented as mean ± SD. **b** Neurofibromin expression in patients-derived tumor xenograft. Scale bar = 50 µm. **c** *NF1* mRNA and protein expression in *NF1*-shRNA#1 and #2-transduced (i) NNI-12 and (ii) NNI-21. HSP90 serves as the loading control. **d** Percentage of invasion in *NF1*-shRNA #1 and #2-transduced NNI-12 and NNI-21. **e** (i) Photomicrographs show H&E staining and neurofibromin expression in shCtrl (*top*) and *NF1*-shRNA#1(*bottom*)-transduced tumor. Black line, invasion zone between tumor and normal; red arrows, pocket of invasive cells; T tumor, N normal. Scale bar = 25 μm. (ii) Photomicrographs show composite of seven images demonstrating the extent of invasion in *NF1*-shRNA-transduced tumor. Red arrow, pocket of invasive cells; black line, invasion zone betwen normal and tumor region. Scale bar = 50 μm. (iii) Quantification of the number of invaded cells in shCtrl and *NF1*-shRNA-transduced tumor. Data shown are absolute value per field (200× original magnification). Student’s *t* test was used to analyze statistical significance between *NF1*-shRNA and shCtrl-transduced tumor, **p < 0.001. **f** Immunohistochemistry staining shows Vimentin, CD44 and Sox2 expression in shCtrl (*top*) and *NF1*-shRNA (*bottom*) transduced tumor. Scale bar = 50 μm. **g** mRNA expression of (i) Vimentin, CHI3L and CD44, and (ii) Sox2 in shCtrl and *NF1*-shRNAs-transduced cells as determined by qPCR. **h** qPCR analysis of EMT markers SNAI1, ZEB2, ZEB1, and TWIST1 in shCtrl and *NF1-*shRNA-transduced cells. **i** Western blot analysis demonstrating expression of p38, STAT3, AKT, p70S6K, S6, SMAD2 in NNI-21 cells transduced with shCtrl and *NF1-*shRNA 72 h post-infection. Actin serves as the loading control. Densitometry quantification was done for the indicated proteins by normalizing to actin. Ratios were indicated below each blot. For (**c**), (**d**), (**g**), and (**h**), data presented are representative from three independent experiments ± SEM. Student’s unpaired *t* test was used to analyze statistical significance between *NF1*-shRNA and shCtrl-transduced cells, *p < 0.01, **p < 0.001, ***p < 0.0001

To assess the expression of *NF1* in GBM subtypes, we analyzed The Cancer Genome Atlas (TCGA) database. *NF1* expression was significantly lower in the more aggressive mesenchymal GBM in comparison to other subtypes as shown in TCGA GBM database analysis (*n* = 203) (Fig. 1a). This finding confirms previous reports that showed *NF1* correlation with mesenchymal GBM [3, 44]. We further supported this finding at the protein level by immunostaining performed using patient-derived GBM tumor sections for neurofibromin expression. Clear nucleus and cytoplasmic staining were observed in the proneural GBM NNI-12 and NNI-21, but were largely absent in the mesenchymal GBM NNI-19 and NNI-24 (Fig. 1b).

Genetic modeling of mesenchymal GBM in mice has been achieved by concurrent deletion or mutation of *NF1*, *p53*, and/or *PTEN* [14, 45, 46]. As such, to investigate the role of *NF1* in glioma invasion in this study, both *p53* and *NF1* transcripts were depleted using shRNAs (Supplemental Fig. 1 and Fig. 1c). *NF1* expression was knocked down using two independent lentiviral shRNAs in two *NF1*-expressing patient-derived glioma propagating cells (GPCs). These GPCs were derived from patient-glioma and characterized as previously described [47–50]. Lentiviral transduction of *NF1*-shRNA-#1 and *NF1*-shRNA-#2 resulted in more than 90% depletion of *NF1* mRNA and protein as confirmed with quantitative polymerase chain reaction (qPCR) and western blot (Fig. 1c). Morphological changes in self-renewal and proliferation after transduction were analyzed by colony forming assay. Depletion of *NF1* resulted in higher self-renewal capability as shown by an increase in the percentage of neurospheres formed in comparison with those of shControl (shCtrl)-transduced cells (Supplemental Fig. 2A). Morphological changes were further evidenced in the higher number of GFP-expressing GPCs (Supplemental Fig. 2B). In addition, these spheres were also larger in size than those of the shCtrl-transduced cells. We further demonstrated that *NF1-*knockdown GPCs proliferate faster than the shCtrl-transduced cells (Supplemental Fig. 2C and D), indicating that *NF1*-loss promotes GPCs self-renewal and proliferation. Of note, the extent of neurosphere formation and clonogenicity was more prominent in *NF1*-shRNA#1.

### NF1 loss promotes GPCs infiltration and exacerbates their ability to invade in vitro and in vivo

We investigated the effect of *NF1* downregulation on invasiveness in *NF1*-positive NNI-12 and NNI-21 GPCs. Increased invasion was observed in both GPCs transduced with the two *NF1*-shRNAs but not with shCtrl lentivirus, indicating that loss of *NF1* promotes cell invasion (Fig. 1d). To evaluate whether *NF1* knockdown induces invasion in vivo, we implanted *NF1*-knockdown NNI-21 GPCs into immunodeficient *NOD*.*SCID* Il2rγ^−/−^ (NSG) mice. In agreement with our in vitro findings, *NF1* silencing markedly increased the number of invasive tumor clusters (red arrows) in comparison to the shCtrl-implanted mice (Fig. 1e(i) and (iii), Supplemental Fig. 3). *NF1*-silenced GPCs colonized the proximal brain hemisphere 9 weeks postimplantation, with invasive cells observed at the parenchyma and invasion zone (Fig. 1e(ii), red arrows). We also tested a panel of three IHC markers which showed differential expression patterns in human proneural vs. mesenchymal glioma (Fig. 1f). These markers, include proneural marker SOX2 [51], and mesenchymal markers vimentin [52], CD44 [53], and chitinase-3-like protein (CHI3L)/YKL40 [44]. Our results showed that *NF1-*knockdown tumor had higher percentage of vimentin and CD44-positive cells (Fig. 1f). Conversely, a lower percentage of SOX2-positive cells was observed in the *NF1-*knockdown tumor (Fig. 1f). Quantification of mesenchymal and proneural markers was performed using qPCR. As expected, higher levels of vimentin, *CHI3L* and *CD44* were observed in *NF1-*shRNAs-transduced cells (Fig. 1g(i)), while *SOX2* mRNA was higher in shCtrl-transduced cells (Fig. 1g(ii)). We additionally investigated whether *NF1*-knockdown promotes EMT by examining changes in EMT markers expression. *NF1* knockdown significantly increased the expression of EMT markers (*SNA1*, *ZEB2*, *ZEB1*, and *TWIST1*) (Fig. 1h), suggesting the activation of EMT-associated signaling pathway. Of the known effectors of *NF1*-loss associated signaling, p38, STAT3, AKT, mTOR, and TGF-β have been previously linked to heightened tumor aggressiveness. We therefore assessed STAT3, AKT, p70S6Kinase, and TGF-β in *NF1-*knockdown cells by measuring their activated forms by western blot (phosphorylated(p)—p38, p-STAT3, p-AKT, p-p70S6Kinase, p-S6, p-SMAD2). p-p38, p-STAT3, p-S6, and p-SMAD2 were marginally increased in *NF1*-knockdown cells, suggesting that these pathways do not play a major role in our system. In stark contrast, levels of p-AKT were low in shCtrl cells, but greatly increased in cells that were transduced with *NF1*-shRNA, suggesting the possible involvement of the AKT signaling pathway (Fig. 1i), supporting our results that *NF1* loss promotes cell proliferation. Collectively, these results demonstrated that *NF1*-loss promotes GBM infiltration and exacerbates their invasive ability.

### NF1-LRD expression reverts cell invasion

**Fig. 2:**
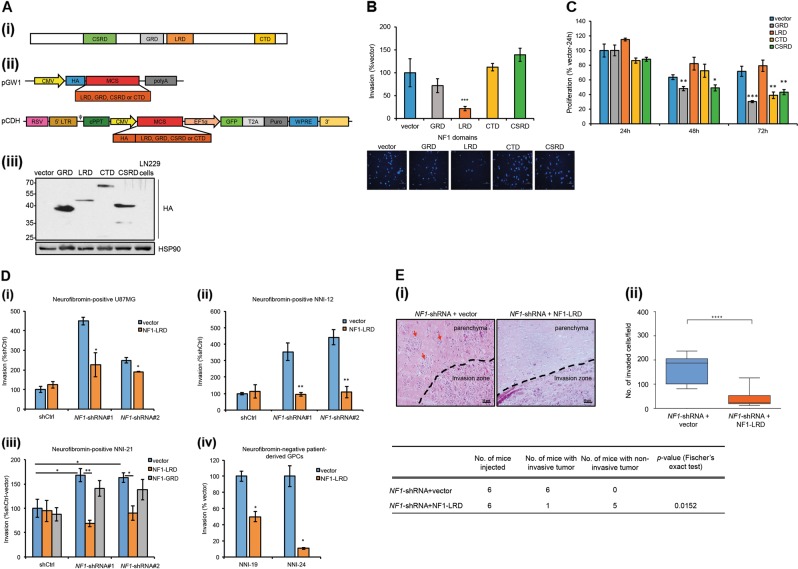
NF1-LRD expression reverts cell invasion. **a** (i) Structure of neurofibromin. CSRD cysteine-serine rich domain, GRD GAP-related domain, LRD leucine-rich domain, CTD C-terminal domain. (ii) Vectors used in this study. The expression vector pGW1 contains the CMV promoter driving transgene expression. Transgene is tagged inframe with HA. pCDH vector is a lentivirus vector-based expression vector. Transgene expression is driven by the CMV promoter. (iii) The four domains were subcloned into pGW1 and transfected into LN229 cells. Immunoblot shows the expression of GRD, LRD, CRD, and CSRD in LN229-transfected cells 48 h post-transfection. Immunoblot was performed using antibody against HA. HSP90 serves as the loading control. **b** Invasion assay was performed on transfected LN229 cells. Percentage of invasion was normalized to that of the vector-transfected cells. Representative images for DAPI-positive cells were shown. *n* = 5 replicates. **c** Effect of proliferation was assessed on LN229-transfected cells at 24, 48, and 72 h post-transfection. Percentage of cell proliferation was normalized to that of vector-transfected cells at 24 h. **d** Invasion assay was performed on (i) U87MG, (ii) NNI-12, (iii) NNI-21 and (iv) neurofibromin*-*negative patient-derived GPCs. **e** (i) Photomicrographs show H&E staining of *NF1-*shRNA vector or NF1-LRD-transduced tumor. Red arrows, pocket of invasive cells; black line, invasion zone between tumor and normal region of the brain. Scale bar = 50 μm. (ii) Quantification of the number of invaded cells in *NF1-*shRNA vector or *NF1-LRD*-transduced tumor per field (200× original magnification). The number of mice with non-invasive tumor in *NF1*-shRNA- NF1-LRD-transduced tumor was summarized in the table. Statistical significance was analyed using Fischer’s exact test. All data are represented as mean ± SEM. Student’s unpaired *t* test was used to analyze statistical significance between NF1-LRD and vector, *p < 0.01, **p < 0.001, ***p < 0.0001, ****p < 0.00001

Neurofibromin contains four major domains, namely CSRD, GRD, LRD, and CTD (Fig. 2a(i)) [54]. To determine whether *NF1*-loss mediated invasion is reversible, we overexpressed these domains of neurofibromin in neurofibromin-deficient LN229 human glioma cells [6]. Because domain-specific antibodies are not available, each domain was tagged with hemagglutinin (HA) for western blot detection (Fig. 2a(ii)). To test the effects of these domains on glioma cell invasion, we transfected the expression plasmids into LN229 cells and assessed the invasion capability. In comparison to the vector control, overexpression of NF1-LRD in LN229 human glioma cells reduced invasion by approximately 80% (Fig. 2b). By contrast, NF1-GRD overexpression reduced invasion by 30%, possibly due to its effect on cell proliferation, while expression of the NF1-CTD and NF1-CSRD domains had no specific effect on cell invasion, suggesting that the cell invasion may be Ras-independent. To further test this, we overexpressed NF1-GRD and NF1-LRD domain in LN229 cells followed by stimulation with recombinant epidermal growth factor (EGF) to assess the Ras-GTPase activity. Remarkably, higher levels of Ras-GTP were observed in NF1-LRD-transfected cells treated with EGF, demonstrating that the NF1-LRD likely played no role in Ras-GAP activity of neurofibromin (Supplemental Fig. 4). We further investigated whether the NF1-LRD played a role in cell proliferation. In comparison to the NF1-GRD where the percentage of proliferation decreased from 24 to 72 h, expression of the NF1-LRD did not inhibit cell proliferation, demonstrating that proliferation activity and invasion capacity were uncoupled (Fig. 2c).

Given that NF1-LRD suppresses cell invasion in neurofibromin-deficient LN229, we were interested in investigating whether NF1-LRD inhibited invasion in *NF1*-expressing glioma cells. U87MG human glioma cells expressing functional neurofibromin were first transduced with *NF1*-shRNAs followed by transduction of either pCDH vector or pCDH-NF1-LRD. In agreement with previous results, knockdown of *NF1* promoted invasion by 2.5–4-fold in comparison to the shCtrl (Fig. 2d(i)). By contrast, re-expression of NF1-LRD inhibited invasion by 25–50% in *NF1-*knockdown cells (Fig. 2d(i)). Similar results were also observed in neurofibromin-expressing NNI-12, whereby re-expression of NF1-LRD suppressed invasion by ~70% (Fig. 2d(ii)). To evaluate whether the effect observed was specific to the NF1-LRD, we transduced *NF1*-knockdown NNI-21 GPCs with NF1-GRD, NF1-LRD, and pCDH vector and compared their invasive capacities. In contrast to NF1-LRD-re-expressing cells, NF1-GRD failed to inhibit *NF1*-loss-induced cell invasion (Fig. 2d(iii)). The percentage of invasion in NF1-GRD-transduced cells was similar to that of the vector control, indicating that the ability to suppress cell invasion was NF1-LRD-specific. We further assessed the effect of re-expressing NF1-LRD in GPCs that did not express neurofibromin (i.e., NNI-19 and NNI-24). As shown in Fig. 2d(iv), NF1-LRD inhibited invasion of NNI-19 and NNI-24 by 50% and 90%, respectively.

To test whether re-expression of NF1-LRD suppresses invasion in vivo, we transduced *NF1-LRD* into *NF1*-knockdown NNI-21 GPCs and assessed the extent of tumor invasion. As shown in Fig. 2e, NF1-LRD significantly inhibited invasion in vivo. In comparison to *NF1*-shRNA vector-transduced cells, clear demarcation between tumor and normal region was observed in NF1-LRD-expressing tumor (Fig. 2e), whereas invasive cells were notably visible in *NF1*-shRNA vector-transduced tumors (red arrows). Quantification carried out to determine the extent of cell invasion showed significantly higher number of invaded cells in vector-transduced *NF1-*knockdown tumor in comparison to those re-expressing NF1-LRD (Fig. 2e(ii)). In fact, five out of six mice implanted with *NF1-LRD*-transduced cells were presented with non-invasive, highly circumscribed tumors when compared with mice implanted with the vector. Taken together, these results demonstrated that NF1-LRD reverts cell invasion.

### NF1-LRD reverses EMT markers expression

**Fig. 3:**
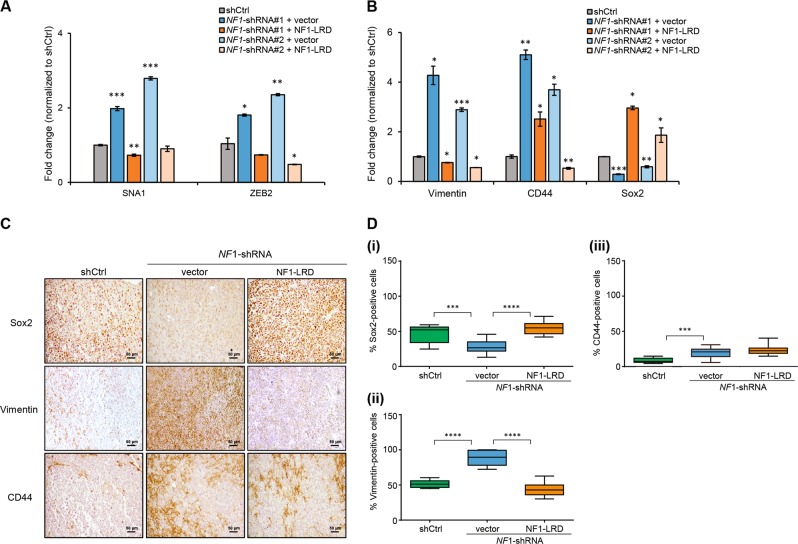
NF1-LRD reverses EMT markers expression. The expression of **a** SNAI1 and ZEB2, **b** *Vimentin*, *CD44*, and *Sox2* were assessed in *NF1*-shRNA-vector or *NF1*-shRNA-NF1-LRD-transduced NNI-21 GPCs. Data shown are fold change of mRNA expression normalized to shCtrl. Data are presented as mean ± SEM. Student’s unpaired *t* test was used to analyze statistical significance between *NF1-LRD* and vector, *p < 0.01, **p < 0.001, ***p < 0.0001. **c** Immunohistochemistry staining of Sox2, Vimentin, and CD44 in *NF1*-shRNA-vector or *NF1*-shRNA-NF1-LRD-transduced NNI-21 tumor. Scale bar = 50 μm. **d** Quantification of (i) Sox2, (ii) Vimentin, and (iii) CD44-positive cells from IHC staining. One-way ANOVA followed by Bonferroni’s post hoc test was performed to analyze statistical significance. *p < 0.01, **p < 0.001, ***p < 0.0001

We previously showed that loss of *NF1* upregulates expression of EMT markers and mesenchymal markers. To assess whether decreased invasion observed in vitro and in vivo is associated with changes in EMT markers expression, we performed qPCR to assess the transcript levels of EMT markers genes *SNAI1* and *ZEB2*. Our results showed lower levels of *SNAI1* and *ZEB2* in *NF1*-shRNA-knockdown cells expressing NF1-LRD when compared with the vector control (Fig. 3a). Similarly, *Vimentin* and *CD44* expression were also lower in *NF1-LRD*-expressing cells, while *SOX2* expression was elevated (Fig. 3b). To rule out the effect of cell culture, we additionally assessed the expression of SOX2, Vimentin and CD44 protein in NF1-LRD-expressing tumor. Similar to qPCR results, a higher percentage of SOX2-positive cells was observed in NF1-LRD-expressing tumor (Fig. 3c, d(i)), while that of Vimentin was lower (Fig. 3c, d(ii)), likely indicating a delayed proneural to mesenchymal transition in the *NF1*-shRNA transduced cells. In contrary to the elevation of *CD44* mRNA transcripts observed in vitro, no significant difference in *CD44* expression was observed between vector and NF1-LRD-expressing *NF1*-shRNA-transduced tumor (Fig. 3c, d(iii)). Taken together, these results suggested that re-expression of NF1-LRD may revert EMT by downregulating EMT-associated transcription factor activity following *NF1* loss.

### Pathogenic mutations in the NF1-LRD domain abolish suppression of invasion

**Fig. 4:**
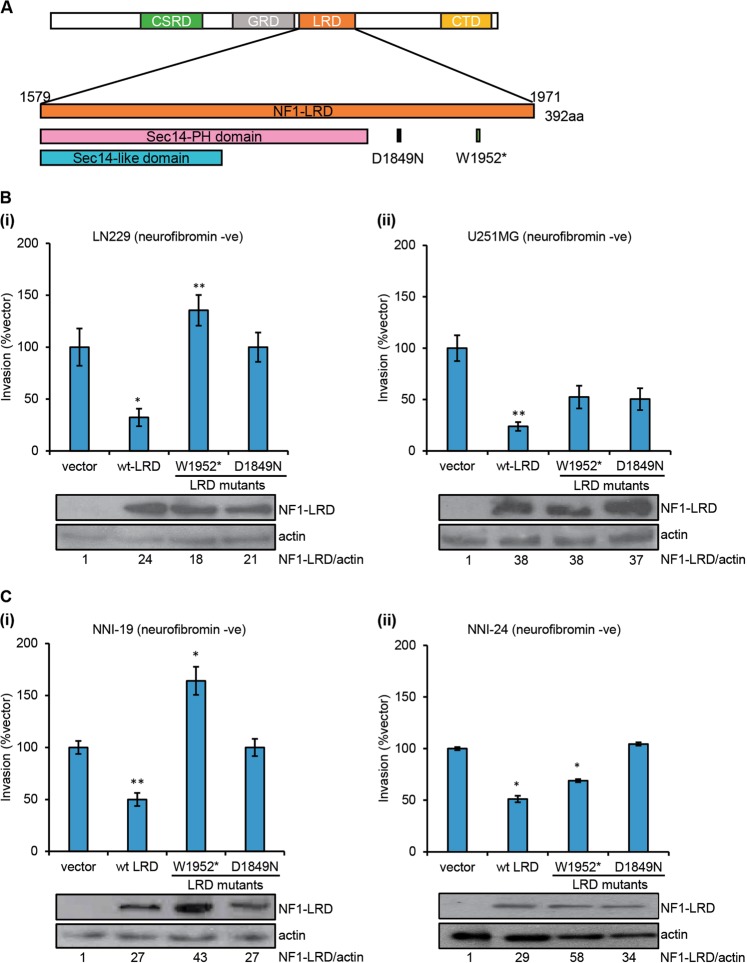
Pathogenic mutations in the NF1-LRD domain abolish suppression of invasion. **a** Oncomap shows the distribution of point mutations in the NF1-LRD domain. Mutations were derived from query performed on cBioportal TCGA dataset. **b** Invasion was assessed in neurofibromin-negative (i) LN229 and (ii) U251MG transduced with pCDH, wt-LRD, W1952* and D1849N. The percentage of invasive cells was normalized to that of pCDH vector. **c** Percentage of invasion was performed on neurofibromiin-negative (i) NNI-19 and (ii) NNI-24 patient-derived GPCs transduced with pCDH, wt-LRD, W1952*, and D1849N. *n* = 4 replicates. Immunoblot demonstrated expression of NF1-LRD as determine by HA expression. Actin serves as loading control. Densitometry quantification was performed by normalizing NF1-LRD expression to actin. Ratios were indicated below the blot. All data are presented as mean ± SEM from three independent experiment, *n* = 5 replicates. Student’s unpaired *t* test was used to analyze statistical significance between wt and mutant LRD to that of vector, *p < 0.01, **p < 0.001, ***p < 0.0001

We then sought to investigate whether mutations, specifically nonsense or missense mutations, found within the NF1-LRD domain will affect the invasiveness of glioma cells. By querying cBioportal TCGA GBM database [11, 12], we identified two mutations within the NF1-LRD domain that were also found in other cancer types such as cutaneous melanoma, colon carcinoma, and infiltrative breast carcinoma (cBioportal TCGA database, Tumor suppressor gene database, NCBI dbSNP, ClinVar, and Human Proteome Variation Database). Using site-directed mutagenesis, we generated two mutants that carry the D1849N (missense) and W1952* (nonsense) mutations (Fig. 4a). We expressed these mutants or wild-type (wt) NF1-LRD in the neurofibromin-deficient LN229 and U251MG cells [6] and compared their invasiveness. Both W1952* and D1849N restored cell invasion in LN229 and U251MG, though to a different extents (Fig. 4b(i) and (ii)). However, enforced expression of NF1-LRD reduced cell invasion by approximately 70% (Fig. 4b(i) and (ii)) in comparison to the vector control. We further examined the effect of NF1-LRD expression in the neurofibromin*-*deficient GBM of mesenchymal subtype from NNI-19 and NNI-24 GPCs (Fig. 4c(i) and (ii)). Consistent with LN229 and U251MG, re-expression of W1952* and D1849N failed to suppress cell invasion when compared to GPC re-expressing NF1-LRD, thus demonstrating that patient-derived mutations abrogate the biological activity of the NF1-LRD domain.

### C-terminal region of NF1-LRD is required for suppressing cell invasion

**Fig. 5:**
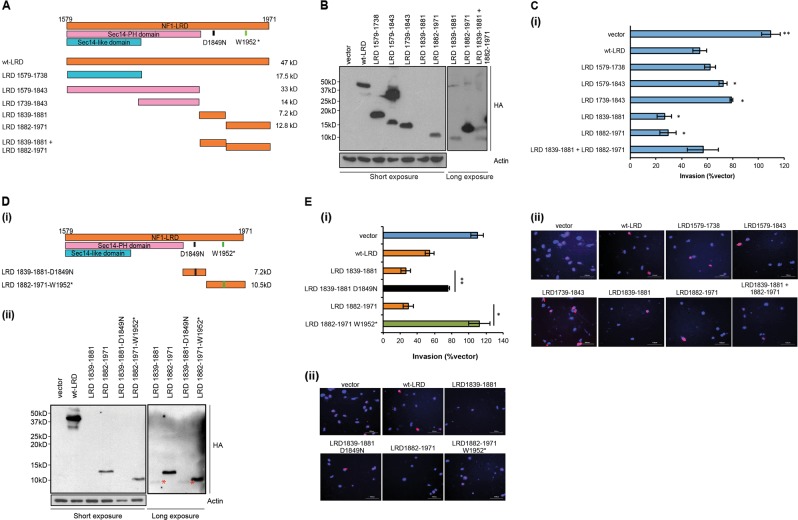
C-terminal region of NF1-LRD domain is required for suppressing cell invasion. **a** Truncation mutants used in this study. **b** Western blot shows the expression of the various truncation mutants as determine by immunblotting for HA. Actin serves as loading control. **c** Invasion assay was performed to determine the region of NF1-LRD that is necessary for suppressing cell invasion. (i) Percentage of invasion was calculated based on the number of HA+ cells vs. HA− cells followed by normalizing to vector-transfected cells. DAPI stained the nucleus. (ii) Representative images showed HA+ cells vs. HA− cells in the membrane. Data are presented as quadruplicate ± SEM. Representative result from five independent experiment was shown. Student’s unpaired *t* test was used statistical analysis, *p < 0.01, **p < 0.001, ***p < 0.0001. **d** (i) D1849N and W1952* mutations were introduced into LRD 1839–1881 and LRD 1882–1971, respectively. (ii) Western blot shows the expression of the mutants as determined by HA. Actin serves as loading control. **e** Effect of point mutations in the peptides LRD 1839–1881 and LRD 1882–1971 was assessed. (i) Percentage of invasion was calculated based on the number of HA + cells vs. HA− cells followed by normalizing to vector-transfected cells. (ii) Representative images showed HA+ cells vs. HA− cells in the membrane. DAPI stained the nucleus. Data are presented as quadruplicate ± SEM. Student’s unpaired *t* test was used statistical analysis, *p < 0.01, **p < 0.001, ***p < 0.0001

We mapped the regions of NF1-LRD that are required for suppressing cell invasion using a series of truncation mutants that span different regions of NF1-LRD (Fig. 5a). These mutants ranged in size from 7 to 33 kDa with similar expression level as shown by HA immunoblotting with the exception of LRD 1839–1881 (7 kDa) (Fig. 5b). We investigated the extent of cell invasion exhibited by these truncation mutants based on the number of HA+ cells vs. HA− cells followed by normalizing to vector-transfected cells. Our results showed that LRD 1579–1738 which contains the SEC14 domain, exhibited comparable level of invasion suppression to wt-LRD. By contrast, the SEC14-PH domain-containing-LRD 1579–1843 and the PH domain-containing-LRD 1739–1843 did not significantly suppress invasion as compared to wt-LRD. Remarkably, LRD 1839–1881 and LRD 1882–1971 displayed the strongest suppression of invasion when compared with the wt-LRD (~80 vs. ~50%; Fig. 5c(i))). It is interesting to note that the expression levels of LRD 1839–1881 were the lowest among the truncation mutants (Fig. 5b), suggesting that this region may be critical for NF1-LRD’s function.

Because D1849N and W1952* mutations fall within LRD 1839–1881 and LRD 1882–1971 fragments, respectively, we employed site-directed mutagenesis to introduce these mutations into both fragments and compared their role in cell invasion (Fig. 5d(i) and (ii)). Our results demonstrate that LRD 1882–1971-W1952*, with a molecular weight of 10.5 kDa (Fig. 5d(ii)), restored cell invasion in comparison to its wt counterpart (Fig. 5e; 112.2 + 12.5% vs. 29.4 ± 6.3%). Similarly, the suppressive effect was lost in cells transfected with LRD 1839–1881-D1849N as opposed to its wt counterpart (% invasion are 75.5 ± 2.12% vs. 26.6 ± 5.7%, respectively, *p* = 0.027; Fig. 5e). Taken together, these results indicated that the LRD region from 1839–1971 is critical for suppressing glioma cell invasion.

## Discussion

Cellular invasion is the key hallmark of GBM that is highly associated with tumor malignancy. Invading tumor cells render complete surgical resection impossible, and recurrence of tumor growth therefore remains an intractable clinical issue. It is believed that glioma cells undergo EMT-like activity that is associated with *NF1* loss or dysregulation. Although *NF1* is one of the most mutated genes and its GRD domain has been studied extensively, very limited is known about its other domains such as NF1-LRD domain in which mutations have been reported in GBM patients. In this study, we demonstrate that the NF1-LRD domain of *NF1* plays a role in glioma invasiveness.

It is known that *NF1* depletion results in hyperactivation of Ras and the subsequent activation of its downstream signaling such as EMT-related transcription factors. Our findings are in agreement with published report [20, 21] whereby loss of *NF1* by shRNA promoted glioma cell invasion and upregulation of EMT markers such as vimentin, SNAI, TWIST1, ZEB1, and ZEB2. By contrast, the proneural marker SOX2 was downregulated, consistent with previous publication in head and neck cancer on the inverse relationship between SOX2 and vimentin [55]. Furthermore, we detected upregulation of p-70S6K and p-Akt in GPCs transduced with *NF1*-shRNA, confirming the observation of Qian et al. [56] that AKT modulates cell migration and invasion through the activation of p-70S6K. In fact, AKT localize at the leading edge of migrating cells [57–60] to enhance actin cytoskeleton rearrangement as well as formation of membrane protrusion required for cell movement [61], and to modulate matrix metalloproteinase-2 and -9 expression [62, 63]. In terms of EMT-associated transcription factors, AKT activates TWIST1, but at the same time, it is also a TWIST1-mediated transcriptional regulator [64]. Notably, we showed that re-expression of NF1-LRD similarly reversed cellular invasiveness in several human glioma cell lines, patient-derived GPCs and an orthotopic mouse glioma model. Consistent with *NF1* knockdown, this NF1-LRD-induced suppression of invasion correlates with increased SOX2 expression and reduced vimentin, SNAI1 and ZEB2 expression. However, re-expression of NF1-LRD suppresses cell invasion but only marginally downregulates p-Akt (Supplemental Fig. 5), suggesting that the NF1-LRD domain likely exerts its influence collaboratively with other neurofibromin domains such as the NF1-GRD, which inhibits proliferation through the RAS/AKT pathway in *NF1*-shRNA transduced cells, or other independent binding partners in the neurofibromin-deficient LN229 and U251MG.

Although we showed that NF1-LRD and its peptides suppress cell invasion, we are cognizant of the limitation of our study is the location of the NF1-LRD domain within neurofibromin and the functional context of NF1-LRD in the full neurofibromin protein. In this study, we defined the NF1-LRD domain as previously reported, i.e., aa 1579–1971 neurofibromin isoform 1 (aa 1558–1950 neurofibromin isoform 2) [35, 41, 54]. This region contains the SEC14-PH domain (aa 1579–1843 neurofibromin isoform 1) and the undefined domain from aa 1839–1971. Aside from the known mutations found within the SEC14-PH domain, the 1839–1971 region harbors several missense mutations that were suggested to be pathogenic (dbSNP and cBioportal) [11, 12, 65]. Based on results from Hsueh’s group [41] and ours, we postulate that this region define the boundary of the NF1-LRD domain. Our assumption is based on the observation that point mutations generated within the NF1-LRD domain (D1849N and W1952*) abolished cell invasion of glioma cells lines and non-NF1-patient-derived glioma cells (Fig. 4). In addition, truncation mutants 1839–1881 and 1882–1971 individually suppressed cell invasion 50% higher than wt-NF1-LRD (Fig. 5). Mutations within these two peptides completely abolished their function, indicating that the region 1839–1971 is functional. It is important to note that although both D1849N and W1952* mutations identified from cancer databases help to dissect the functional significance of NF1-LRD in GBM, they are nevertheless different from mutations identified from NF1 patients who may have increased susceptibility to NF1-associated glioma.

The LRD 1839–1971 peptide region coincides with a portion of the HLR. The HLR is composed of two linked alpha helices that are structurally related to the coiled coil domain of ARM repeats that are similarly found in Plakoglobin, also known as γ-catenin. In breast cancer cells, Plakoglobin is involved in the translocation of E-cadherin and β-catenin to inhibit cell invasion [66–69]. While the functionality of the NF1-LRD has yet to be elucidated in the context of full neurofibromin protein, it has been hypothesized that NF1-LRD may function as a scaffold protein to interact with phospholipids for membrane localization owing to its SEC14 domain [42] that is adjacent to the GAP domain and the PH-like domain that associate with proteins involved in signal transduction [70]. In addition, the HLR domain may also function in protein–protein interaction. Since the induction of the invasive phenotype requires cooperation between multiple factors, it is possible that the NF1-LRD may independently interacts with other proteins to bring about its suppressive effect. Future work may entail the elucidation of the 3D structure of NF1-LRD and the full neurofibromin protein to decipher its active binding region as well as conformation. Currently, the crystal structure of NF1-LRD is not available due to its poor stability even with the used of codon-optimized constructs [71]. However, this issue may be overcome with the use of the cloning-friendly *NF1* mini-genes by the Morrison’s group [72], which may also aid in identification of the potential interaction partner(s).

In summary, our results show, for the first time, the NF1-LRD domain reverts *NF1*-loss induced invasion, and provides initial evidence into the otherwise novel function of NF1-LRD in glioma biology.

## Materials and methods

### Cell culture and transfection

This study was approved by the Centralized Institutional Review Board of SingHealth (Singapore). Patient-derived GPCs are kind gifts from Drs Ang BT and Tang C (National Neuroscience Institute, Singapore) after informed consent. A total of four GPCs isolates were used in this study; these are NNI-12, NNI-21, NNI-19, and NNI-24. The GPCs were cultured in Dulbecco’s Modified Eagle’s Media (DMEM)/F12 (Sigma-Aldrich, Inc., MO, USA) supplemented with B27 (Invitrogen, Carlsbad, CA), EGF (R&D Systems, Minneapolis, MN), basic fibroblast growth factor (R&D Systems) and heparin (Sigma-Aldrich, Inc.). Human glioma cell lines U87MG, U251MG and LN229 were obtained from American Type Cell Culture (ATCC, Manassas, VA) and authenticated in 2017 and confirmed mycoplasma negative by service provider. These cells were cultured in DMEM with 10% fetal bovine serum (FBS) (Invitrogen).

For in vitro transfection, 2–12 µg of plasmid DNA were transfected into 0.3–2.5 × 10^6^ human glioma cells using Viafect™ (Promega, Madison, WI) or polyethylenimine, linear, MW 25000 (PEI) (Polysciences, Inc., Warrington, PA) in accordance with the manufacturer’s instructions.

### Plasmids

shCtrl and *NF1*-shRNA with the pGFP-shLenti backbone were obtained from OriGene (OriGene, Rockville, MD). The plasmids, HA-LRD, HA-GRD, HA-CSRD, and HA-CTD, in pGW1 backbone, are kind gift from Dr Hsueh YP (Academia Sinica, Taiwan). To generate deletion mutants of NF1-LRD, sequences encoding for the various mutants were synthesized (Integrated DNA Technologies, Inc., Coralville, IA) and subcloned into *Bgl*II site of pGW1-HA. All sequences were verified by DNA sequencing (1st BASE, Singapore). Refer to Supplementary methods for full method.

### Invasion assay

Invasion assay was performed using Corning® Matrigel® Invasion Chamber according to manufacturer’s instructions (Corning Incorporated, Corning, NY). Briefly, GPCs or glioma cells (5 × 10^4^) suspended in either DMEM/F12 medium or DMEM containing 5% FBS were added to the top chamber. DMEM containing 10% FBS was added the bottom chamber. The extent of invasion was quantified after 24 h by counting the number of invasive cells at the underside of the chamber at original magnification x200. All assays were performed in quadruplicates, and images from five random fields were taken for each replicate. Refer Supplementary methods for full method.

### Quantitative real-time PCR

Quantitative real-time PCR was performed as described previously [73]. Expression levels of the various targets were quantified using LightCycler 96 (Roche Holding AG, Basel, Switzerland). All qPCR reactions were performed in duplicate. Expression level was calculated using the ddCT method. The relative expression level was calculated by arbitrarily designating the lowest normalized value to 1.

### Immunoblotting

Proteins were extracted from cells in RIPA buffer (10 mM Tris pH 7.4, 1× IGEPAL, 0.5% sodium deoxycholate, 0.1% sodium dodecyl sulfate) containing phosphatase and protease inhibitor (Roche). For detection of neurofibromin expression, denatured protein samples were resolved in 3–8% tris-Acetate gel (Invitrogen) followed by transferred onto a 0.45 µm polyvinylidene fluoride membrane (Merck & Co., Kenilworth, NJ), which was then blocked in 5% milk in Tris-buffered saline containing 0.1% Tween-20 and probed with the required antibodies. Membranes were blotted against the following antibodies: NF1(D), NF1 (H-12), and β-actin (C4) (Santa Cruz Biotechnology, Inc., Dallas, TX); phospho-p38, total p38, phospho-STAT3 (Ser727), total STAT3, phospho-AKT (Ser473), total AKT, phospho-p70S6K (Thr389), total p70S6K, phospho-S6 ribosomal protein, total S6, HA-Tag (C29F4), pSMAD2, total SMAD2, and HSP90 (Cell Signaling Technology, Inc., Danvers, MA). List of antibodies used are shown in Table 1. Refer to Supplementary methods for full methods.

**Table 1:** List of antibodies used

Protein target	Manufacturer and cat. no.
β-Actin (C4)	Santa Cruz sc-47778
Neurofibromin (H-12)	Santa Cruz sc-376886
Neurofibromin (D)	Santa Cruz sc-67
HA-Tag (C29F4)	Cell Signaling #3724
Phospho-Akt (Ser473)	Cell Signaling #9271
Akt	Cell Signaling #9272
Phospho-Stat3 (Ser727)	Cell Signaling #9134
Stat3 (79D7)	Cell Signaling #4904
Phospho-p70 S6 Kinase (Thr389) (1A5)	Cell Signaling #9206
p70 S6 Kinase	Cell Signaling #9202
Phospho-p38 MAPK (Thr180/Tyr182) (D3F9) XP^®^	Cell Signaling #4511
p38 MAPK	Cell Signaling #9212
Phospho-S6 ribosomal protein (Ser235/236) (D57.2.2E) XP^®^	Cell Signaling #4858
S6 ribosomal protein (5G10)	Cell Signaling #2217
Phospho-SMAD2 (pSer465/467)	Merck #566415
SMAD2/3 (C-8)	Santa Cruz sc-133098
HSP90	Cell Signaling #4874
CD44std (SFF-304)	eBioScience™ BMS150
Vimentin (D21H3) XP^®^	Cell Signaling #5741
SOX2	R&D Systems MAB2018
Anti-mouse IgG, HRP-linked	Cell Signaling #7076
Anti-rabbit IgG, HRP-linked	Cell Signaling #7074

### Immunofluorescence and immunohistochemistry staining

Immunohistochemistry analysis was performed on 5 µm thick paraffin-embedded 4% PFA fixed tissue sections with the following primary antibodies diluted in PBS containing 0.1% Tween-20, 3% goat serum and 0.1% bovine serum albumin: CD44std (SFF-304, 1:50 dilution, eBiosciences, Thermo Fisher Scientific), SOX2 (Clone #245610, 1:300 dilution, R&D Systems Inc.), NF1 (D) (sc-67, 1:100 dilution), and Vimentin (D21H3, 1:25 dilution, Cell Signaling Technology, Inc.). Target protein expression was detected using DAB followed by counterstaining with hematoxylin and visualized with an inverted microscope (Eclipse TE2000-S, Nikon, Japan) at 20×/0.45 numerical aperture (N.A.) Plan Fluor objective (Nikon) or 60× objective. Images were quantified from 8–10 sections using ImmunoRatio, an ImageJ (NIH) plugin that uses deconvolution algorithm to separate and quantify nuclear staining using DAB [74]. For immunofluorescence staining, cells fixed with 4% PFA was incubated with anti-HA antibody followed by Alexa-Fluor 594 conjugated secondary antibody incubation. All immunofluorescence-stained samples were examined under confocal microscope (FV1000, Olympus, Japan) at 20×/0.75 N.A. UPlanSApo objective (Olympus). Refer to Supplementary methods for full method.

### In vivo tumor implantation

All animal experiments were performed in accordance to the guidelines and protocols approved by the Institutional Animal Care and Use Committee at National Neuroscience Institute, Singapore. Inoculation of tumor cells in immunodeficient mice was performed as previously described [75]. GPCs were preinfected with multiplicity of infection 50 of either shCtrl, *NF1*-shRNA, pCDH, or NF1-LRD viral vectors. Transduced cells (5 × 10^5^ cells) were implanted into the right hemisphere (Bregma 0,0, lateral 2 mm, anterior 1 mm, depth −2.5 mm) of six male immunodeficient NSG mice (InVivos, Singapore) of 6- to 8-week old. The number of animals injected with the tumor cells were determined based on our experience, where 6 mice are sufficient to demonstrate approximately 50% difference in tumor growth. Glioma-bearing mice were sacrificed 2 months post-tumor implantation when neurological deficit such as cachexia, hunched-back, and lethargy was observed. No randomization was used. Investigators were not blinded during analysis.

To assess the extent of tumor cells invasion, mice were perfused through the heart with ice-cold saline followed by 4% paraformaldehyde (PFA). Mice brains were harvested and kept in 4% PFA overnight at 4 °C, transferred to 30% sucrose in PBS for additional 48 h prior to paraffin embedding or cyrosectioning.

### Statistical analysis

All results were presented as mean ± SEM with the exception of Fig. 1a. Figure 1a was presented as mean ± S.D. All in vitro experiments were performed in three to eight replicates and were repeated at least thrice independently. Statistical analysis was performed using Prism 6.0 (Graphpad Software, Inc., La Jolla, CA). One-way analysis of variance followed by Tukey–Kramer or Bonferroni multiple comparisons test were used for comparing statistical significance for more than two groups. Unpaired two-sided Student’s *t* test was used for comparing between two groups of equal variance. *p* Value < 0.05 was considered statistically significant.

## Supplementary information

Supplemental information

Supplemental information

Supplemental Fig. 1

Supplemental Fig. 2

Supplemental Fig. 3

Supplemental Fig. 4

Supplemental Fig. 5
