# Supplementary material for: Pathogenic mutations in neurofibromin identifies a leucine-rich domain regulating glioma cell invasiveness
Source: Oncogene. 2019 Apr 9;38(27):5367–80. doi: 10.1038/s41388-019-0809-3 (PMC6755990; doi:10.1038/s41388-019-0809-3)
Supplement: Supplementary file 2 — Supplemental information [file 41388_2019_809_MOESM2_ESM.docx]

**Supplemental information**

Supplemental information includes supplemental methods, supplemental figure legends, and supplemental figures.

**Supplemental methods**

***Plasmids***

Non-targeting shRNA (shCtrl) and *NF1*-targeted shRNAs (*NF1*-shRNA) with the pGFP-shLenti backbone were obtained from OriGene (OriGene, Rockville, MD). The plasmids, HA-LRD, HA-GRD, HA-CSRD and HA-CTD, in pGW1 backbone, are kind gift from Dr Hsueh YP (Academia Sinica, Taiwan). pGW1 vector was generated by excising LRD from HA-LRD using *Bgl*II, followed by religation. To subclone these plasmids into pCDH lentivirus vector, primers harboring restriction enzyme sites *Bst*BI, *Nhe*I and *Sal*I were generated. HA-LRD, HA-GRD, HA-CSRD and HA-CTD were PCR amplified using respective PCR primers and subcloned into pCDH vector. Site-directed mutagenesis (Agilent Technologies, Santa Clara, CA) was carried out using the primers 5’-acacaccaagattcggccaaaaaatgtccctggga-3’ and 5’-gtttggaatacatgactccatagctgtcaaatctagttcgt-3’ for inserting p.D1849N and p.W1952* mutation, respectively, into pCDH-LRD. To generate deletion mutants of LRD, sequences encoding for the various mutants were synthesized (Integrated DNA Technologies, Inc., Coralville, IA) and subcloned into *Bgl*II site of pGW1-HA. All sequences were verified by DNA sequencing (1^st^ BASE, Singapore).

***Invasion assay***

Invasion assay was performed using Corning® Matrigel® Invasion Chamber according to manufacturer’s instructions (Corning Incorporated, Corning, NY). Briefly, GPCs or glioma cells (5 × 10^4^) suspended in either DMEM/F12 medium or DMEM containing 5% FBS were added to the top chamber. DMEM containing 10% FBS was added the bottom chamber. Following 24h incubation, non-invading cells were removed from the top chamber using cotton swabs. The invaded cells on the underside of the membrane were fixed with 4% PFA and stained with 4',6-diamidino-2-phenylindole (DAPI) (Sigma-Aldrich, Inc.). The invaded cells were counted using an inverted microscope at original magnification x200. The cell invasion assay was done in quadruplets, and five fields were randomly taken for each membrane.

***Immunoblotting***

Proteins were extracted from cells in RIPA buffer (10 mM Tris pH 7.4, 1x IGEPAL, 0.5% Sodium deoxycholate, 0.1% SDS) containing phosphatase and protease inhibitor (Roche). Quantification of total protein was carried out using Protein Assay Dye Reagent (Bio-Rad Laboratories, Inc., Hercules, CA) and the absorbance reading at wavelength 595 nm was obtained using Tecan Infinite M200 Microplate Reader. Denatured protein samples were resolved in 10% Tris-Glycine gel, 3-8% Tris-Acetate gel (Invitrogen) and 16% Tris-Glycine gel (Bio-Rad Laboratories) and transferred onto a polyvinylidene fluoride (PVDF) membrane (Merck & Co., Kenilworth, NJ), which was then blocked in 5% milk in Tris-buffered saline (TBS) containing 0.1% Tween-20 and probed with the required antibodies. Membranes were blotted against the following antibodies: NF1(D) and NF1 (H-12) and β-actin (C4) (Santa Cruz Biotechnology, Inc., Dallas, TX); phospho-p38, total p38, phospho-STAT3 (Ser727), total STAT3, phospho-AKT (Ser473), total AKT, phospho-p70S6K (Thr389), total p70S6K, phospho-S6 ribosomal protein, total S6, HA-Tag (C29F4), pSMAD2, total SMAD2, and HSP90 (Cell Signaling Technology, Inc., Danvers, MA). The specific proteins of interest were detected on autoradiography films (Santa Cruz Biotechnology, Inc.) using SuperSignal™ West Pico PLUS Chemiluminescent and SuperSignal™ West Femto Maximum Sensitivity substrates (Thermo Fisher Scientific, Waltham, MA). When required, the blots were stripped with agitation in Restore™ PLUS Western Blot stripping buffer (Thermo Fisher Scientific) for 5-10 min to re-probe another primary antibody of interest. ImageJ software (NIH, Bethesda, MA) was used for analysis of band densitometry.

***Ras Activation Assay***

Cells were serum starved overnight before harvesting for Ras activation assay. The next day, cells were incubated with recombinant EGF for 5 min prior to harvesting. Ras activity assay was performed using Active Ras pull-down and detection kit (Thermo Fisher Scientific) according to manufacturer’s instructions. Fresh lysates containing 500 µg of total proteins from each cell lines were incubated with 80 µg of Raf-1 RBD agarose beads for 3h at 4°C. GTPγS and GDP were loaded to serve as positive and negative control, respectively. The precipitated GTP-Ras was eluted in 2x SDS sample buffer and visualized by immunoblotting with anti-Ras antibodies. The level of Ras activation was then expressed as a ratio of Ras-GTP to the total Ras/actin levels in the same lysates.

***Immunofluorescence and immunohistochemistry staining***

For immunohistochemistry analysis, 5 µm thick paraffin-embedded tissue sections were incubated with the following primary antibodies: CD44std (SFF-304, eBiosciences, Thermo Fisher Scientific), SOX2 (Clone #245610, R&D Systems Inc.), NF1 (D) and Vimentin (D21H3, Cell Signaling Technology, Inc.). Secondary antibody incubation utilised the horseradish peroxidase-conjugated anti-rabbit/mouse polymer, followed by detecting with 3,3'-diaminobenzidine (Dako^TM^ Envision + Dual Link System – HRP, Agilent Technologies) and counterstaining with hematoxylin. Sections were visualised with an inverted microscope (Eclipse TE2000-S, Nikon, Japan) at 20×/0.45 numerical aperture (N.A.) Plan Fluor objective (Nikon) and 60x objective. To quantify the percentage of positive cells, images were analysed using ImageJ (NIH) plugin called ImmunoRatio (University of Tampere, Finland), which uses deconvolution algorithm to separate and quantify nuclear staining using DAB [64]. We obtained the immunopositive staining of sections using automated cell counting in 8-10 sections at original magnification x200.

For immunocytochemistry staining, the cells were first seeded onto coverslips in 24-well plate, fixed with 4% PFA, and blocked with 5% goat serum. Primary incubation of anti-HA and secondary incubation with Alexa Fluor 594 were carried out. All immunofluorescence-stained samples were examined under confocal microscope (FV1000, Olympus, Japan) at 20×/0.75 N.A. UPlanSApo objective (Olympus). Negative control was included in all the immunostaining, where primary antibody was omitted.

**Supplemental figure legends**

**Supplemental Fig. 1**

Quantification of p53 knockdown was assessed in NNI-12 and NNI-21 GPCs transduced with shCtrl and p53-shRNA 72h post-infection. Data presented are + SEM. Student's unpaired *t*-test was used statistical analysis, *p<0.01, **p<0.001, ***p<0.0001.

**Supplemental Fig. 2**

Colony forming assay was performed in shCtrl and *NF1-*shRNA-transduced NNI-12 and NNI-21 patient-derived GPCs. (A) Percentage of neurospheres formed was normalized to shCtrl-transduced cells. (B) GFP images showed the neurospheres size. Data are presented as quadruplicate + SEM. (C) and (D) Cell proliferation of *NF1-*shRNAs and shCtrl-transduced NNI-12 (C) and NNI-21 (D) GPCs. Cells were transduced with the respective shRNAs and control. Cell proliferation was analyzed using CCK8 (a tetrazolium dye reduction method) assay at different timepoints. The OD450nm at various time points were normalized to the respective samples at Day 1. N=6 replicates. Student’s unpaired *t*-test was used to analyze statistical significance among the samples. *p<0.05. *p<0.01, **p<0.001, ***p<0.0001.

**Supplemental Fig. 3**

Photomicrograph shows the H&E images of shCtrl and *NF1*-shRNA-transduced NNI-21 tumor. T, tumor; N, normal; black line, invasion zone between normal and tumor; red arrows, pocket of invasive cells. Scale bar= 25μm.

**Supplemental Fig. 4**

Ras-GTPase assay was performed to determine the RAS-GAP activity of NF1-LRD. pGW1 vector, NF1-GRD or NF1-LRD transfected cells were serum starved overnight before harvesting for Ras activation assay. The next day, cells were stimulated with recombinant EGF for 5 min prior to harvesting. Cells were immunoblotted using anti-Ras antibody. Actin serves as loading control.

**Supplemental Fig. 5**

Akt activation was determined in (A) LN229 and (B) U251MG cells transfected with vector and wt-LRD. Densitometry quantification was performed by normalizing the p-Akt to total Akt. Ratios were indicated below each blot.
