# Supplementary figures and images for: Pathogenic mutations in neurofibromin identifies a leucine-rich domain regulating glioma cell invasiveness
Source: Oncogene. 2019 Apr 9;38(27):5367–80. doi: 10.1038/s41388-019-0809-3 (PMC6755990; doi:10.1038/s41388-019-0809-3)

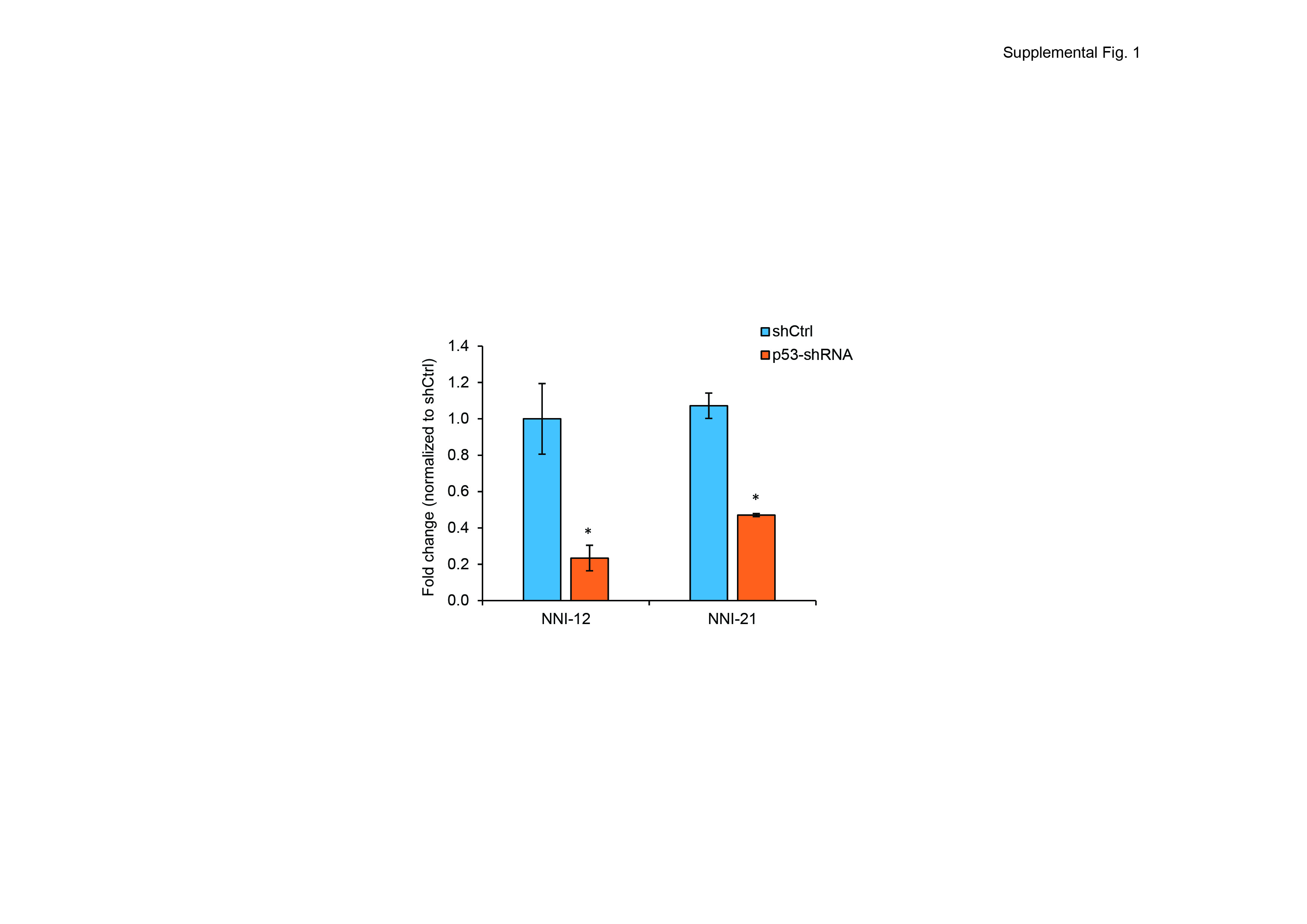

Supplement: Supplementary file 3 — Supplemental Fig. 1 [file 41388_2019_809_MOESM3_ESM.jpg]

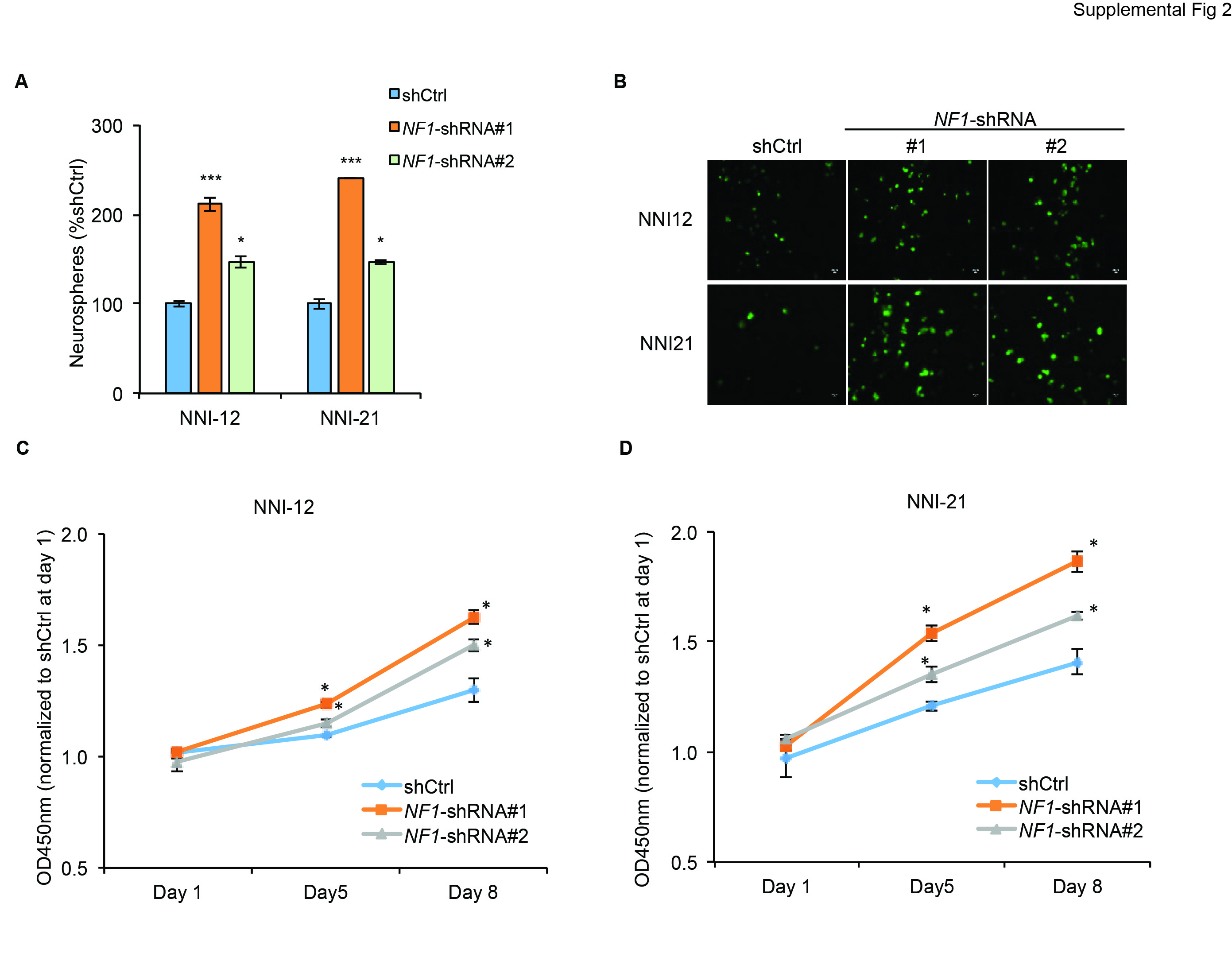

Supplement: Supplementary file 4 — Supplemental Fig. 2 [file 41388_2019_809_MOESM4_ESM.jpg]

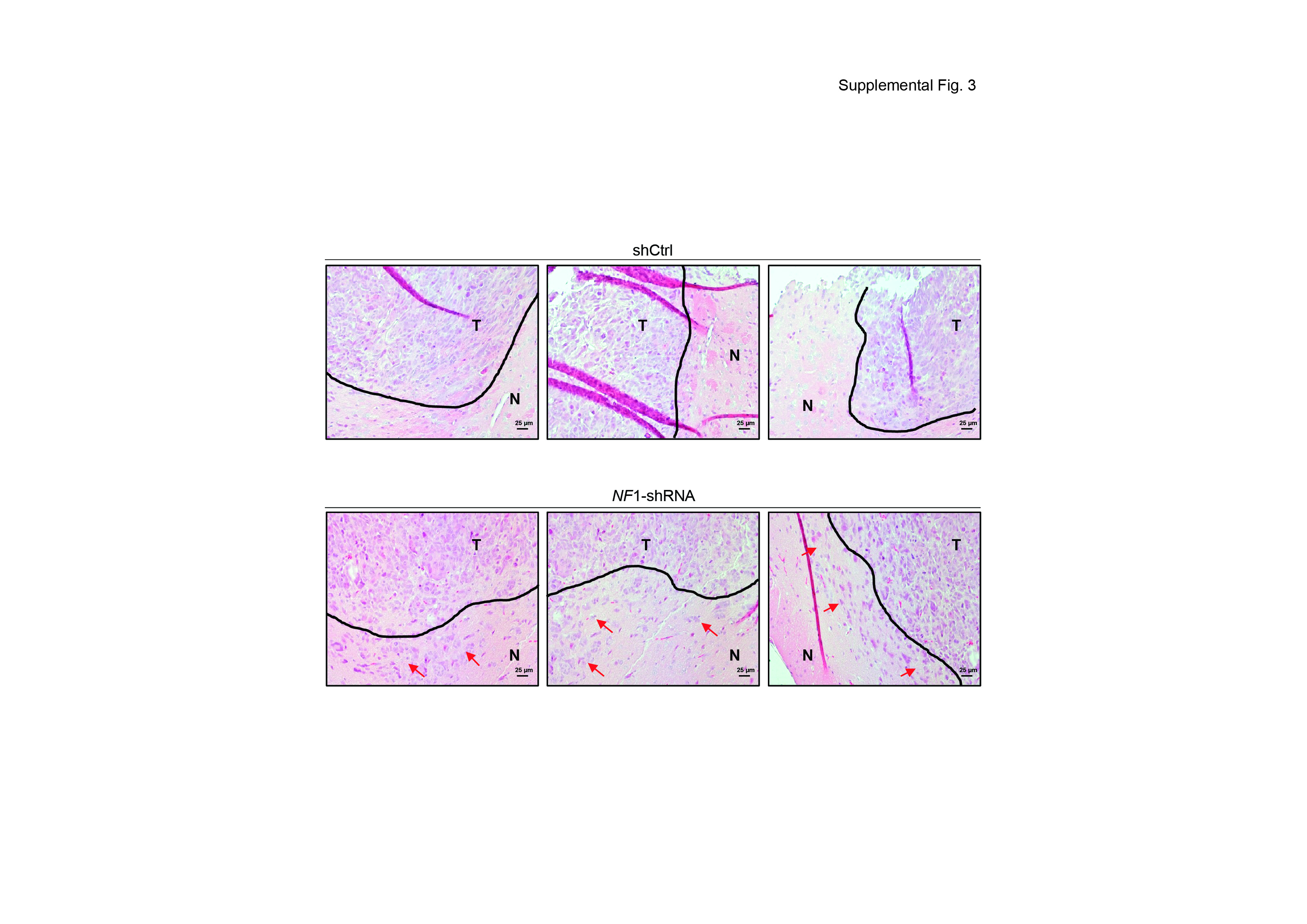

Supplement: Supplementary file 5 — Supplemental Fig. 3 [file 41388_2019_809_MOESM5_ESM.jpg]

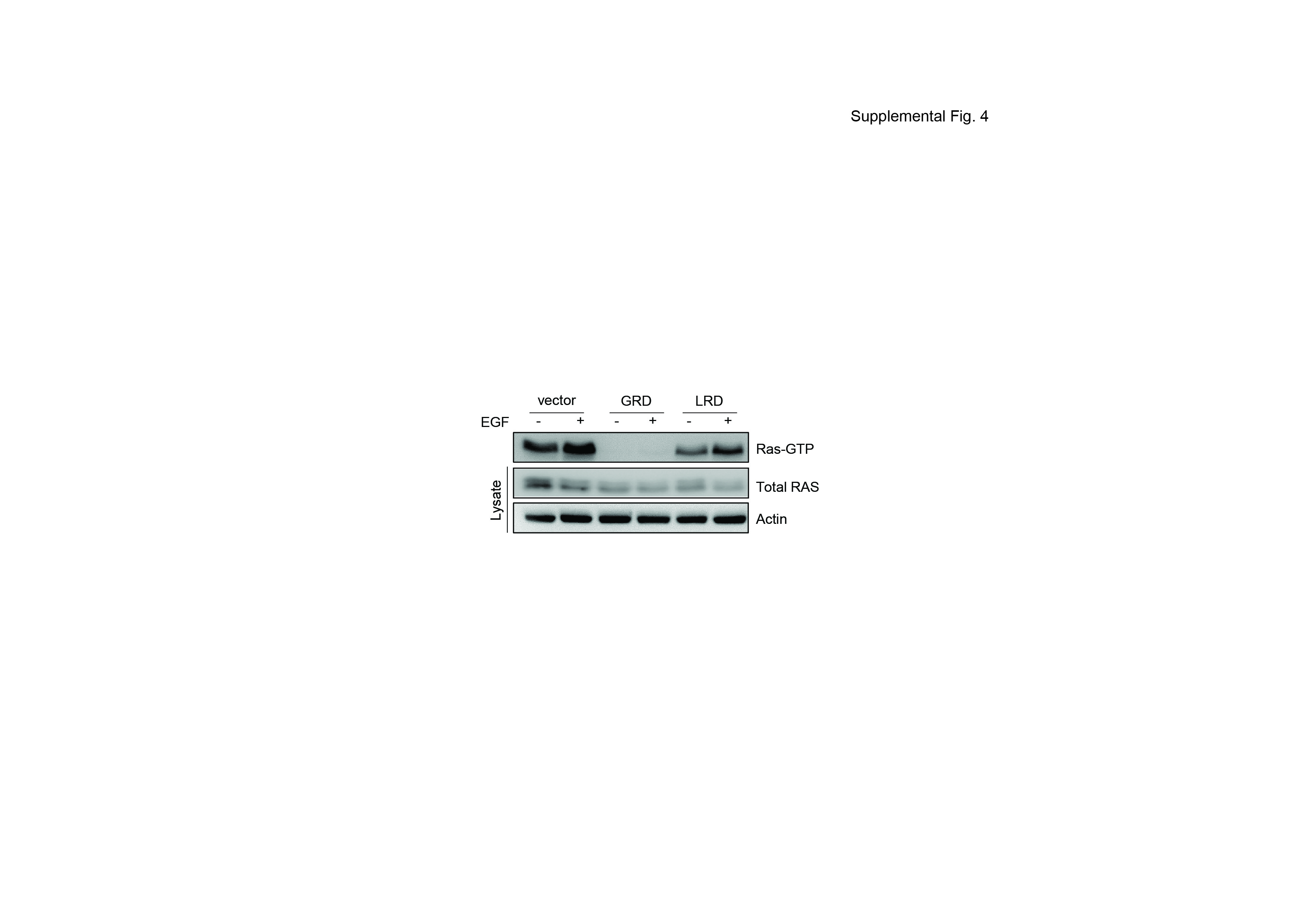

Supplement: Supplementary file 6 — Supplemental Fig. 4 [file 41388_2019_809_MOESM6_ESM.jpg]

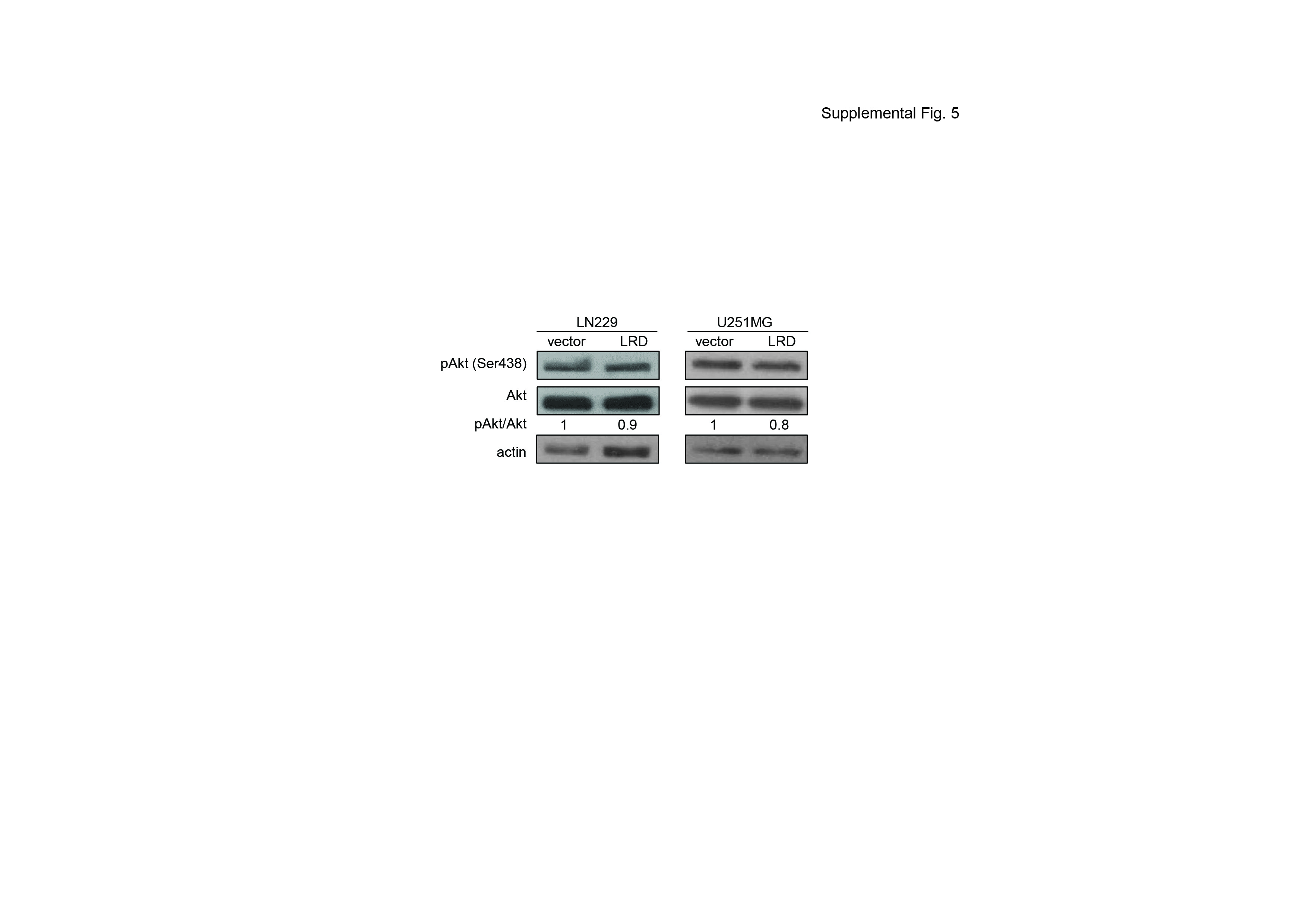

Supplement: Supplementary file 7 — Supplemental Fig. 5 [file 41388_2019_809_MOESM7_ESM.jpg]
